# High Throughput Selection of Effective Serodiagnostics for *Trypanosoma cruzi* infection

**DOI:** 10.1371/journal.pntd.0000316

**Published:** 2008-10-08

**Authors:** Gretchen Cooley, R. Drew Etheridge, Courtney Boehlke, Becky Bundy, D. Brent Weatherly, Todd Minning, Matthew Haney, Miriam Postan, Susana Laucella, Rick L. Tarleton

**Affiliations:** 1 Center for Tropical and Emerging Global Diseases and Department of Cellular Biology, University of Georgia, Athens, Georgia, United States of America; 2 Instituto Nacional de Parasitología “Dr. Mario Fatala Chabén”, Buenos Aires, Argentina; Institut Pasteur, France

## Abstract

**Background:**

Diagnosis of *Trypanosoma cruzi* infection by direct pathogen detection is complicated by the low parasite burden in subjects persistently infected with this agent of human Chagas disease. Determination of infection status by serological analysis has also been faulty, largely due to the lack of well-characterized parasite reagents for the detection of anti-parasite antibodies.

**Methods:**

In this study, we screened more than 400 recombinant proteins of *T. cruzi*, including randomly selected and those known to be highly expressed in the parasite stages present in mammalian hosts, for the ability to detect anti-parasite antibodies in the sera of subjects with confirmed or suspected *T. cruzi* infection.

**Findings:**

A set of 16 protein groups were identified and incorporated into a multiplex bead array format which detected 100% of >100 confirmed positive sera and also documented consistent, strong and broad responses in samples undetected or discordant using conventional serologic tests. Each serum had a distinct but highly stable reaction pattern. This diagnostic panel was also useful for monitoring drug treatment efficacy in chronic Chagas disease.

**Conclusions:**

These results substantially extend the variety and quality of diagnostic targets for Chagas disease and offer a useful tool for determining treatment success or failure.

## Author Summary

The diagnosis of *Trypanosoma cruzi* infection (the cause of human Chagas disease) is difficult because the symptoms of the infection are often absent or non-specific, and because the parasites themselves are usually below the level of detection in the infected subjects. Therefore, diagnosis generally depends on the measurement of *T. cruzi*–specific antibodies produced in response to the infection. However, current methods to detect anti–*T. cruzi* antibodies are relatively poor. In this study, we have conducted a broad screen of >400 *T. cruzi* proteins to identify those proteins which are best able to detect anti–*T. cruzi* antibodies. Using a set of proteins selected by this screen, we were able to detect 100% of >100 confirmed positive human cases of *T. cruzi* infection, as well as suspect cases that were negative using existing tests. This protein panel was also able to detect apparent changes in infection status following drug treatment of individuals with chronic *T. cruzi* infection. The results of this study should allow for significant improvements in the detection of *T. cruzi* infection and better screening methods to avoid blood transfusion–related transmission of the infection, and offer a crucial tool for determining the success or failure of drug treatment and other intervention strategies to limit the impact of Chagas disease.

## Introduction

Chagas disease, a consequence of infection by the protozoan parasite *Trypanosoma cruzi*, affects up to 20 million individuals primarily in the Americas where the insect vectors are present and where zoonotic transmission cycles guarantee a steady source of parasites. *T. cruzi* infection has its greatest human impact in areas of Latin America where housing conditions bring people, infected animals, and vector insects into close proximity [1]. In addition, increasing travel and immigration has brought *T. cruzi* infection into the spotlight globally, even in areas where transmission has previously been absent or very low. In these latter situations, congenital and transfusion/transplantation-related transmissions are becoming recognized as significant threats [2],[3],[4].

Diagnosis of *T. cruzi* infection is challenging for a number of reasons. The initial infection is seldom detected except in cases where infective doses are high and acute symptoms very severe, as in localized outbreaks resulting from oral transmissions [5],[6],[7]. Classical signs of inflammation at proposed sites of parasite entry (e.g. “*Romaña*'s sign”) or clinical symptoms other than fever, are infrequently reported [8]. As a result, diagnosis is very rarely sought early in the infection, when direct detection of parasites may be possible. In the vast majority of human cases, *T. cruzi* infection evolves undiagnosed into a well-controlled chronic infection wherein circulating parasites or their products are difficult to detect even with the use of amplification techniques. A “conclusive” diagnosis of *T. cruzi* infection is often reached only after multiple serological tests and in combination with epidemiological data and (occasionally) clinical symptoms.

Unfortunately, multiple studies from geographically distinct areas and utilizing a wide range of tests and test formats have shown current diagnostics to be far from dependable [9],[10],[11],[12],[13],[14],[15],[16],[17],[18],[19] Many of the most widely employed serological tests, including one recently licensed by the United States Food and Drug Administration for use as a blood screening test in the U.S. [20], use crude or semi-purified parasite preparations, often derived from parasite stages present in insects but not in infected humans. Other tests have incorporated more defined parasite components, including multiple fusion proteins containing epitopes from various parasite proteins, which, individually have shown some promise as diagnostics [13],[21],[22]. Unfortunately, in the absence of a true gold standard, the sensitivity of new tests is generally determined using sera that have been shown to be unequivocally positive on multiple other serologic tests, but rarely with sera that are borderline or equivocal on one or more tests. This approach assures only that the test being evaluated is no worse, but not necessarily any more sensitive, than the existing tests.

In this study, we sought to improve upon current diagnostics for *T. cruzi* infection by screening for diagnostic candidates that displayed the ability to detect infection in subjects that went undetected or gave discordant results using other conventional serologic tests. The end result is the identification of a panel of recombinant proteins that more reliably detects *T. cruzi* infection than do a combination of existing conventional tests. Additionally, we show that a multiplex assay utilizing this diagnostic panel has utility in monitoring drug treatment efficacy in chronic Chagas disease.

## Methods

### Parasites, gene selection and cloning

*T. cruzi* epimastigotes of the SylvioX10/4 (original source human, Brazil), CL Brener (insect, Brazil), Brazil (human, Brazil), CL, Tulahuen (insect, Chile), M83, M91 (both from human, Argentina) and Chapulin (dog, Argentina) isolates were maintained in logarithmic phase growth [23] and used as a source of genomic DNA. Primer sets incorporating lambda phage recombination sites flanking the 18–21 base gene-specific sequence (excluding both start and stop codons), were designed for each gene of interest, the genes cloned by PCR from the pooled DNA of the 8 *T. cruzi* isolates, and Gateway adapted gene product inserted into the pDONR-201 plasmid (Invitrogen, Carlsbad, CA). To speed the cloning process while improving our chances of cloning full-length non-mutated genes, at least 10 clones positive for the appropriate sized insert were pooled for each gene. *T. cruzi* lysate from Brazil strain amastigote and trypomastigotes was prepared as previously described by 4 freeze-thaw cycles followed by sonication [24].

### pDEST-PTD4 Construction

The pDEST-PTD4 protein expression vector was created from pTAT-HA [25] by replacing the BamHI-flanked TAT sequence with a BamHI-flanked PTD-4 encoding sequence [26], followed by Gateway (Invitrogen)- adaptation of the plasmid utilizing the NcoI and XhoI cloning sites. The Gateway cloning cassette was PCR amplified from pDEST-YFP (gift from Dr. Boris Striepen, University of Georgia, GA).

### Protein production and purification

Genes in pDONR plasmids were transferred to pDEST-PTD4 via a Gateway LR reaction and the proteins expressed in BL21(DE3)pLysS cells were extracted by sonication in 8 M urea, 20 mM HEPES, 100 mM NaCl, pH 8.0 containing 15 mM imidazole. The lysate was then applied to TALON Metal Affinity Resin (BD Biosciences Clonetech, Palo Alto, CA) and bound protein was eluted with 250 mM imidazole. Imidazole was removed on PD-10 desalting columns (GE Healthcare, Piscataway, NJ) and protein concentration was estimated using a modified Bradford assay. Proteins were diluted to 10 µg/mL (in 8 M urea) and stored in 1 mL aliquots at −20 C until use.

### Human Sera

Sera were obtained from individuals living in areas of Santiago del Estero, Argentina endemic for *Trypanosoma cruzi and* were analyzed using conventional serologic tests (e.g. immunofluorescence assay (IFI), hemagglutination (HAI), and ELISA to detect IgG [27]) performed at the Diagnostic Department of the Instituto Nacional de Parasitología “Dr. Mario Fatala Chabén” and in our laboratory by a commercial ELISA serodiagnostic kit (Hemagen Diagnostics, Columbia, MD). The latter assay was carried out as per the manufacturer's instructions with a positive response defined as 10% above the cutoff (0.250+mean of negative control absorbencies). Three serum pools were created: a “sero-negative” pool consists of 4 sera negative on all assays; a “borderline positive” pool made up of 5 sera with a response at or just above the equivocal zone of the Hemagen test (between cutoff and below cutoff+10%); a “strong positive” pool containing 7 sera that gave unequivocally positive responses on all tests. True negative controls (16 total) were obtained from volunteer donors who were not from endemic areas (North America and Buenos Aires). Sera (from 175 total subjects) used for subsequent analysis of individual proteins were obtained from *T. cruzi*-infected adult volunteers aged 29 to 61 recruited through the Chagas Disease Section of the Cardiology Department, Hospital Interzonal General de Agudos “Eva Perón”, Buenos Aires, Argentina and infection status was determined serologically as described above. In some cases, subjects treated by a 30 day course of benznidazole as previously described [28] donated serum samples prior to treatment and at regular intervals following treatment. In all cases, sera were collected from clotted blood obtained by venapuncture, then aliquoted and stored at −70°C and subjected to a minimum possible number of freeze/thaw cycles. The protocols were approved by the IRBs of the University of Georgia and the Hospital Interzonal General de Agudos “Eva Perón” and signed informed consent was obtained from all individuals prior to inclusion in the study.

### Multiplex Assay

Recombinant proteins were attached to Liquichip Ni-NTA beads (Qiagen) or Beadlyte Nickel Beads (Upstate Biotechnology) by incubating excess protein (10 ug/ml) with an equal volume of beads overnight at 4°C in the dark. The sets of distinct addressable beads, each with a different protein attached, were pooled in equal volumes along with positive and negative control beads, consisting respectively of Liquichip Carboxy Beads (Qiagen) coupled to *T. cruzi* lysate and Liquichip Ni-NTA beads coated with recombinant HIS-tagged enhanced green fluorescent protein (EGFP). Sera at 1∶500 dilutions were added and the multiplex assays conducted using standard procedures [29]. Antibody binding to individual beads was detected with donkey anti-human IgG (H+L) conjugated to phycoerythrin (cat no. 709-116-149, Jackson ImmunoResearch, West Grove, PA) and quantified on a BioPlex Suspension Array System (BioRad).

### Statistical Analysis

Serum samples were assayed in duplicate and the weighted mean fluorescence intensity (MFI) was calculated for a minimum of 30 beads per determination. The ratio of the specific MFI for each antigen to the MFI of the negative control (GFP- or OVA-coupled) protein was then calculated for each serum and antigen in the assay. Values above the mean plus 4 standard deviations of a minimum of sixteen true negative sera run in the same assay, and individually determined for each antigen, were considered positive.

## Results

As part of a vaccine discovery effort, nearly 1500 genes from *T. cruzi* have been cloned into Gateway entry vector plasmids that allow them to be easily moved into a range of other plasmids. Genes were selected for cloning using a variety of criteria, initially including known expression in *T. cruzi* lifecycle stages that are present throughout infection in mammals (i.e. trypomastigotes and amastigotes), high likelihood of being surface expressed or secreted and expected presence in the genome at low copy number. With the completion of the *T. cruzi* genome sequencing project [30] and whole organism proteome analysis [31] the additional criterion of being relatively high in abundance in the proteomes of trypomastigotes and amastigotes was added as a basis for selection. Recombinant proteins produced in *E. coli* had N-terminal tags carrying the 6XHis-, PTD [26] and HA- tags for purification, protein translocation, and identification, respectively were captured by Ni-coupled Luminex beads for use in a multiplex bead array assay. The Luminex platform is based upon the use of internally stained and individually addressable bead sets which can be coupled with different molecules (in our cases, *T. cruzi* proteins) and then used to measure the concentration of multiple analytes (antibodies specific for the individual proteins) in a solution (serum). Identification of the distinct beads and quantitation of antibody binding is accomplished by flow cytometry. As many as 100 different analytes can be simultaneously assayed in <100 ul sample.

### Selection of the diagnostic panel

**Figure 1:**
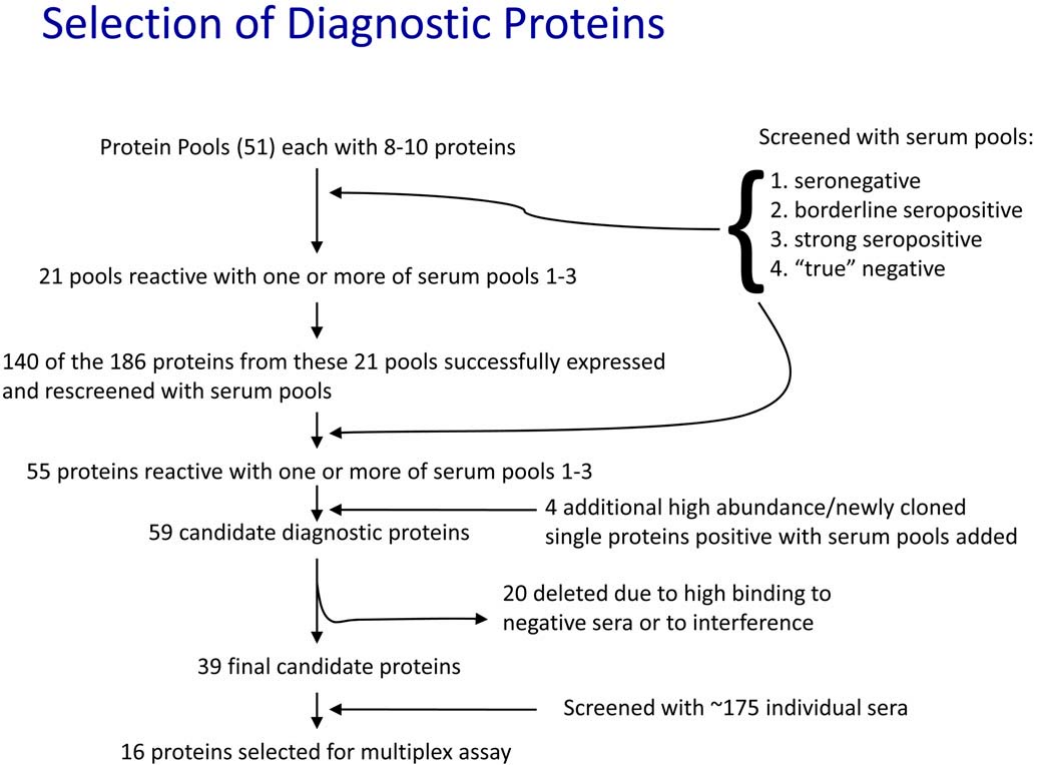
Screening process for the high-throughput selection of diagnostic proteins for detection of *T. cruzi* infection.

**Figure 2:**
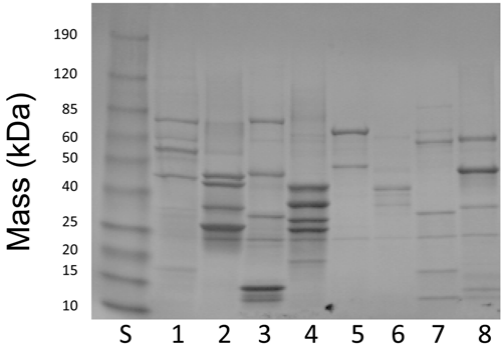
SDS-PAGE gel of production of pooled protein. Sets of 6–8 genes were moved in pools from pDONR entry plasmids into pDEST-PTD4 via a Gateway LR reaction (Invitrogen) and the resulting plasmids transformed into BL21(DE3)pLysS cells for protein production. Recombinant HIS-tagged proteins were purified on Co+2 affinity resin and the bound proteins analyzed by SDS-PAGE. Protein pools depicted in lanes 1–5 were generated from the pooling of 8 genes, while lanes 6, 7 and 8 were derived from 7, 6, and 6 genes respectively. Lane “S” contained molecular weight standards (BenchMark Prestained Protein Standard; Invitrogen). Overall, approximately 80% of genes yielded proteins when expressed as pools.

The initial selection screen (**Figure 1**) used approximately 420 proteins produced in pools of 8–10 proteins each. Production of pooled proteins was accomplished by moving sets of genes in batch into the PTD-4 expression plasmid and was confirmed by SDS-PAGE analysis (**Figure 2**). In addition to the individual or pooled recombinant *T. cruzi* proteins, each screening experiment included negative control recombinant protein (ovalbumin or GFP) expressed from the PTD-4 plasmid as well as a lysate of trypomastigotes and amastigotes of *T. cruzi* that had been chemically coupled to BioPlex beads.

To screen the pooled proteins we also took a pooling approach by assembling sera from subjects with and without documented infection with *T. cruzi*. Screening of 51 protein pools revealed 21 pools that were reactive with one or more of the serum pools 1–3 (**Figure 1**). Reactive pools were then broken down into their individual constituent proteins; a total of 140 proteins were successfully expressed and individually rescreened with the serum pools, ultimately resulting in the selection of 55 proteins with serodiagnostic potential (**Figure 1** and **Table 1**). An additional 22 proteins that were either identified as high-abundance proteins using proteome analysis [31] and/or as being unique to *T. cruzi* (and thus not encoded in the *T. brucei* or *Leishmania major* genomes) were then screened using the pooled sera, and 4 of these 22 were found to be reactive with one or more serum pools. Of the resulting 59 candidate proteins recognized by antibodies in the serum of *T. cruzi*-infected subjects, 20 were subsequently excluded from further testing either because they exhibited significant reactivity with sera from the true negative pool, or because they interfered with other beads in the multibead assays, perhaps because of protein-protein interactions. Preference was also given to *T. cruzi* proteins that detected antibodies in sera from the “borderline” pools. Ultimately 39 proteins (in bold in **Table 1**) were selected for extensive further testing with a wider array of individual subject sera.

**Table 1:** The 59 candidate diagnostic proteins screened independently with individual (non-pooled) sera.

Gene Id	Gene name(s)	Notes	% reactive with 121 known positive sera
**Tc00.1047053506391.10, Tc00.1047053509233.180**	**calmodulin and ATPase beta subunit**	high abundance	32.23%
**Tc00.1047053507029.30**	**heat shock 70 kDa protein, mitochondrial precursor, putative**	high abundance	52.89%
**Tc00.1047053510955.40**	**axoneme central apparatus protein, putative**		42.15%
**Tc00.1047053511215.119**	**69 kDa paraflagellar rod protein, putative**		23.97%
**Tc00.1047053511271.10**	**dispersed gene family 1 fragment 4**	unique to *T. cruzi*	5.08%
**Tc00.1047053506529.610**	**hypothetical protein**		17.27%
**Tc00.1047053506391.30**	**EF-hand protein 5**		2.48%
**Tc00.1047053506635.130**	**hypothetical protein, conserved**	high abundance	68.60%
**Tc00.1047053511265.10**	**dispersed gene family 1 fragment 5**	unique to *T. cruzi*	8.62%
**Tc00.1047053511289.30**	**aminopeptidase, putative**		11.57%
**Tc00.1047053506195.110**	**malate dehydrogenase, putative**	high abundance	24.79%
**Tc00.1047053508461.140**	**poly(A)-binding protein**	high abundance	34.17%
**Tc00.1047053508441.20**	**glycosomal phosphoenolpyruvate carboxykinase, putative**	high abundance	59.29%
**Tc00.1047053508355.250**	**60S acidic ribosomal subunit protein, putative**	high abundance	75.21%
**Tc00.1047053511633.79**	**microtubule-associated protein homolog**	high abundance	74.38%
**Tc00.1047053510433.20 Tc00.1047053504277.11 Tc00.1047053504157.130**	**TolT proteins**	unique to *T. cruzi*	74.38%
**Tc00.1047053411235.9**	**alpha tubulin**		
**Tc00.1047053510877.30**	**hypothetical protein, conserved**		
**Tc00.1047053509695.220**	**serine carboxypeptidase (CBP1), putative**		
**Tc00.1047053510887.50**	**hypothetical protein, conserved**		
**Tc00.1047053509141.40**	**hypothetical protein, conserved**		
**Tc00.1047053506247.220**	**histidine ammonia-lyase**		
**Tc00.1047053509995.10**	**60S ribosomal protein L4, putative**		
**Tc00.1047053504163.50**	**fructose-bisphosphate aldolase, glycosomal, putative**		
**Tc00.1047053507089.270**	**dihydrolipoyl dehydrogenase, putative**		
**Tc00.1047053511019.90**	**iron superoxide dismutase, putative**		
**Tc00.1047053509017.20**	**hypothetical protein, conserved**		
**Tc00.1047053506529.360**	**cytochrome C oxidase subunit IV, putative**		
Tc00.1047053510187.50	tyrosine aminotransferase, putative		
Tc00.1047053505989.110	hypothetical protein, conserved		
Tc00.1047053508209.140	protein disulfide isomerase, putative		
**Tc00.1047053506531.20**	**hypothetical protein, conserved**		
Tc00.1047053504153.280	hypothetical protein, conserved		
Tc00.1047053509233.180	ATPase beta subunit, putative		
**Tc00.1047053506563.40**	**beta tubulin**		
**Tc00.1047053506459.290**	**elongation factor-1 gamma, putative**		
Tc00.1047053508707.200	nucleoside diphosphate kinase, putative		
**Tc00.1047053506529.460**	**hypothetical protein, conserved**		
**Tc00.1047053506297.270**	**60S ribosomal protein L28, putative**		
**Tc00.1047053511527.34**	**60S ribosomal protein L2, putative**		
**Tc00.1047053507483.4**	**polyubiquitin, putative**		
Tc00.1047053509053.70	p22 protein precursor, putative		
**Tc00.1047053506585.40**	**glucose-regulated protein 78, putative**		
Tc00.1047053511185	dispersed gene family 1 fragment 8		
Tc00.1047053511589.130	14-3-3 protein, putative		
Tc00.1047053511167.90	14-3-3 protein, putative		
Tc00.1047053507241.30	arginine kinase, putative		
Tc00.1047053510579.70	nascent polypeptide associated complex subunit, putative		
Tc00.1047053506925.300	cyclophilin a		
**Tc00.1047053509775.40**	**iron superoxide dismutase, putative**		
**Tc00.1047053503583.40**	**trans-splicing factor, putative**		
Tc00.1047053510099.120	d-isomer specific 2-hydroxyacid dehydrogenase-protein, putative		
Tc00.1047053507093.300	hypothetical protein, conserved		
Tc00.1047053508479.340	succinyl-CoA synthetase alpha subunit, putative		
Tc00.1047053509815.120	dispersed gene family 1 fragment 9		
Tc00.1047053511727.270	RNA-binding protein, putative		
Tc00.1047053503781.80	universal minicircle sequence binding protein (UMSBP), putative		
Tc00.1047053506201.39	translation elongation factor 1-beta, putative		
**Tc00.1047053506815.20**	**hypothetical protein**		

Note: Tc00 numbers indicate closest homologue(s) present in the *T. cruzi* CL Brener sequence database (TcruziDb.org) based upon sequencing of the genes (for top 16) or predicted based upon primer sequences used in cloning. Because some primers for PCR cloning were designed prior to the release of the *T. cruzi* CL Brener sequence (25) and the cloning involved the pooling of multiple clone derived from the PCR of a mixture of *T. cruzi* strains (see Material and Methods), some proteins were derived from mixtures of genes (e.g. numbers 1 and 16) and/or had a percent sequence identity <100% relative to the CL Brener strain (range 94.7 to 100%). In some cases (e.g. # 5 and 9) genes>2 kb in length were cloned in ∼2 kb fragments in order to facilitate cloning and protein production. Items listed in bold type were selected for screening using >100 individual sera. Items underlined were selected to be part of the final 16 set bead array for screening of discordant sera or sera from subjects post-treatment with benznidazole.

**Figure 3:**
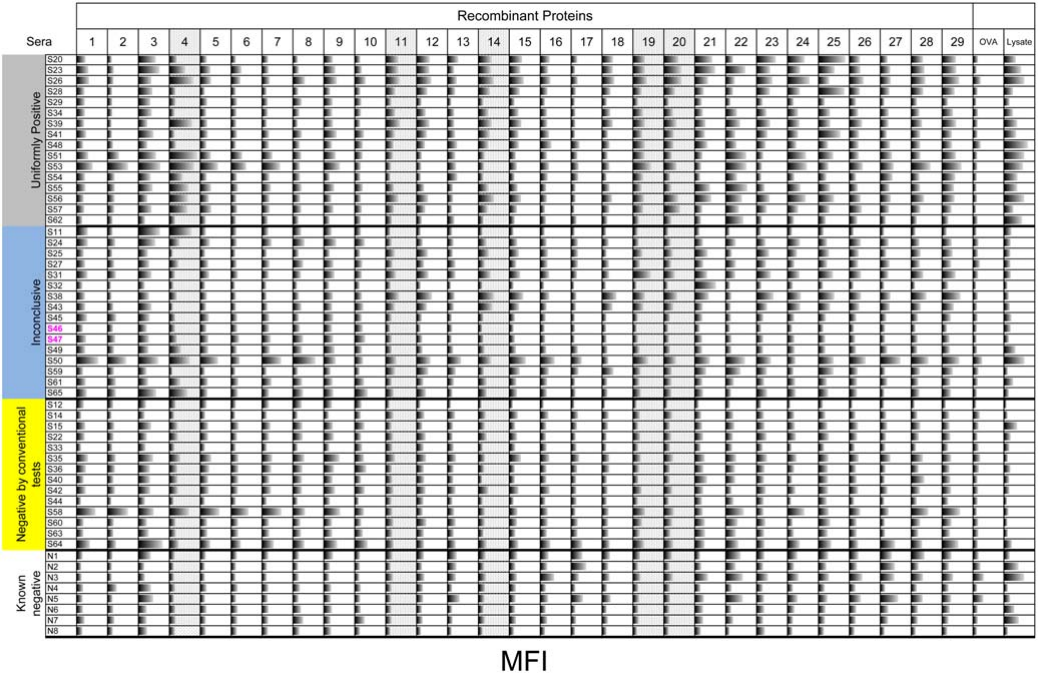
Reactivity of a representative set of proteins tested with individual sera. A selection of 29 individual recombinant proteins was tested for the ability to bind IgGs present in the sera of 54 subjects. The sera are grouped as “uniformly positive” (reactive on all three conventional serological tests and a commercial assay kit), “inconclusive” (negative on at least one conventional serologic tests), “negative by conventional tests” (negative by all three conventional tests), and “known negative” (from residents of North America with very low chance of being infected based upon residency and travel history). Recombinant ovalbumin and *T. cruzi* lysate –coated beads were used as negative and positive controls, respectively. Horizonal bars in each box indicate mean fluorescence intensity (MFI) on a scale from 0 to 30,000 arbitrary light units. A number of the recombinant proteins either failed to discriminate between uniformly positive and known negative sera sets (e.g. 3, 16, 17, 22, 26, 27, 28) or showed no reaction with either set (e.g. 10). In contrast a number of proteins detected nearly the entire uniformly positive group, as well as some in the inconclusive and conventional negative groups but none in the known negative set (e.g. 4, 11, 14, 19 and 20 – stippled highlighting).

Although the Luminex bead array technology theoretically accommodates up to 100 distinct, addressable beads in a single well – and thus the ability to assay up to 100 individual proteins - at the time of this work only 17 distinct beads were available with the ability to capture his-tagged proteins. Thus our goal in the second part of the screen was to identify a set of the 16 best *T. cruzi* proteins (allowing a bead for a control non-*T. cruzi* protein). The 39 candidate diagnostic proteins were tested in sets of 8–15, with each protein on a separate bead and with a negative control bead (HIS-tagged ovalbumin (OVA)) and a positive control bead (*T. cruzi* lysate) included in each assay sample. Between 38 and 48 individual sera from endemic subjects were used to test each protein. These sera are grouped as “uniformly positive” (reactive on all conventional serological tests), “inconclusive” (positive on at least one, but not all, conventional serologic tests), and “negative by conventional tests”, and “known negative” (from residents of North America). **Figure 3** shows a representative set of 29 proteins tested with 54 individual sera and indicates the range of reactivities of both sera and proteins. In addition to providing the basis on which to select the top proteins, this analysis also revealed that among the 30 sera that were inconclusive or negative on conventional tests, nearly half (14 of 30) had substantial reactivity to 3 or more recombinant *T. cruzi* proteins but not with the control OVA protein.

Following repeated screening, 16 proteins were selected to be part of the diagnostic panel (underlined in **Table 1**). DNA sequencing and mass spectrometric analysis confirmed the identity of each gene and protein and determined that one of the preparations contained two distinct proteins (calmodulin and an ATPase) and a second contained a mixture of related TolT proteins. This protein set was then used to screen a larger set of sera, most from chronically infected subjects living in Buenos Aires, and the percentage of these proteins reactive with 121 sera from well-characterized subjects was determined (**Table 1**). A serum was determined to be positive for any particular test antigen if the average luminescence (MFI) was >4 standard deviations above that of a set of true negative sera run in the same assay. Across all experiments, for the 19 true negative sera assayed multiple times (142 sample runs tested on 16 protein preparations for a total of 2272 determinations), none had S.D.>4 and only 17 of the 2272 determinations were >3 S.D. above the average negative serum values (and 9 of these 17 were from one serum sample reactive with the same antigen in multiple tests). Thus, this was a highly stringent cutoff. Sera from all 121 of the confirmed chronically infected subjects reacted with at least 1 of the 16 recombinant protein preparations at the >4 S.D. cutoff and all but 7 reacted with >1 protein. As shown in **Table 1**, 6 of the 16 of the antigens each detected >50% of the sera and 3 antigens approached a 75% detection rate. Of the 121 sera tested, 118 would have been detected as positive using only 4 of the antigens and 100% would be detected using as few as 7 antigens.

### Borderline samples

**Figure 4:**
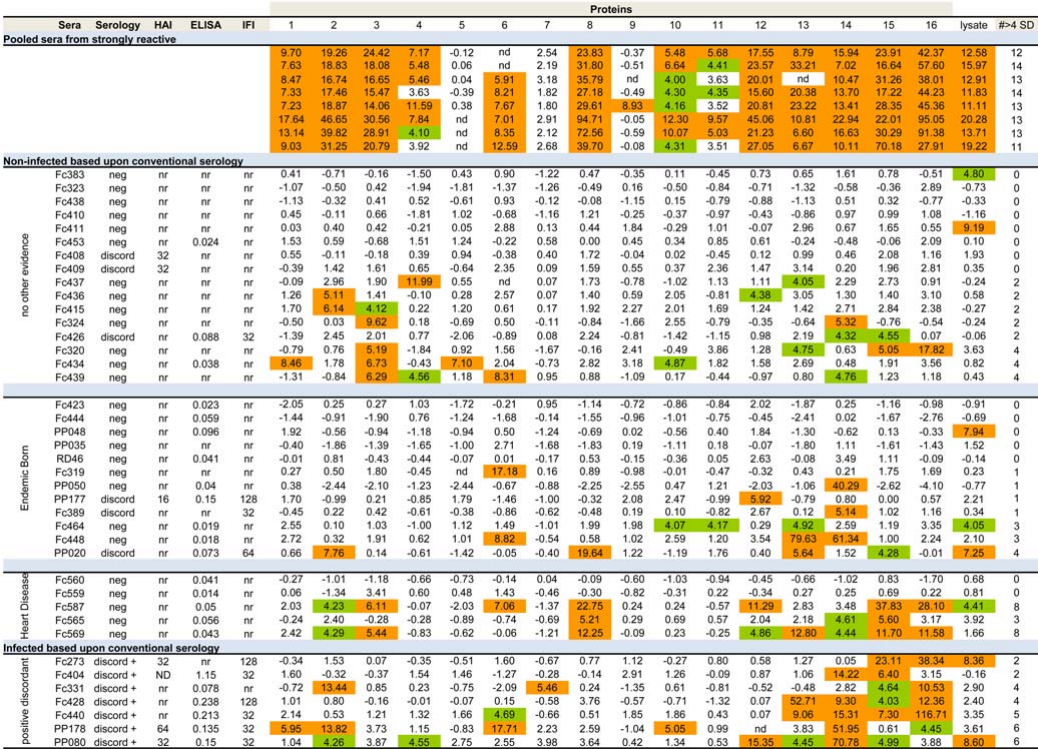
Reactivity of negative, borderline or discordant sera in the 16 protein multiplex assay. Sera judged cumulatively as “seronegative” based upon conventional serology were grouped into negative but “no other evidence” of exposure (16 sera), those “born in an endemic area” (12), those with evidence of “heart disease” consistent with Chagas disease (5) and compared to (top) pools of strongly positive sera (high reactivity in all serological tests) and to (bottom) sera from subjects who were negative on at least one of the three conventional serologic tests (discordant positive). Reactivity in the conventional serological tests (HAI, ELISA and IFI) and the summary consensus of these tests (neg = below cut-off for all three tests; discord = positive on one of the three tests; discord + = positive on 2 of the 3 tests), as well as reactivity to the 16 recombinant protein sets and the *T. cruzi* lysate are shown. Cutoffs for a positive ELISA is an O.D.>0.200 and for IFA and HAI is a dilution>1/32 (a reaction at 1/16 is considered “reactive but negative” and <1/16 non-reactive (nr). The metric for reactivity of each serum for each protein is expressed as the number of standard deviations that the ratio of the MFI for *T. cruzi* protein to the MFI for GFP was above the average ratios of sixteen true negative sera run in the same assay. Values>4 S.D. above this “background” reactivity are considered reactive and are colored. The total number of reactive recombinant proteins for each serum is indicated in the right-most column. nd = not determined (insufficient numbers of beads detected in this sample).

We then used our 16 bead multiplex test to attempt to resolve questionable infection status in subjects due to discordant results on conventional tests (**Figure 4**). In this analysis, a cutoff for reactivity for each protein in the panel was set at the MFI plus 4 SD above the mean of a set of 16 negative sera. For comparison, the result of multiplex analysis of a pool of strongly positive sera assayed on different days is also shown. The strong positive serum pool showed excellent cross-assay consistency with 11 of the 16 protein preparations positive on each of 8 assays and consistent negative reactivity with 3 of 16. Antibodies to the remaining 2 proteins were also detected but at a lower level that sometimes fell below the strict cutoff of 4 S.D. above the mean. The sera classified as “conventional seronegative with no other evidence of infection” broke into 2 groups based upon the results of the multiplex test. Eight of the 16 failed to react with any of the 16 protein panel (although several reacted with the *T. cruzi* lysate) while the remaining 8 reacted with 2–4 proteins. A similar nearly 50/50 split was observed in the group of 12 conventional seronegatives who were born in an endemic region, and in 5 individuals who had cardiopathologies consistent with Chagas disease. Lastly, testing in the multiplex assay of sera classified as “positive discordant” (based upon reactivity on 2 of the 3 conventional serologic tests but negative on the 3^rd^ test) confirmed the positive diagnosis in all 7 cases with reactivity evident on 2–6 recombinant proteins by each serum. Without a clear gold standard diagnostic it is not possible certify on a case-by-case basis that the multiplex assay more accurately detects infection than does conventional serology - particularly in cases where there is reactivity to only 1 or 2 proteins and near the >4 S.D. cutoff. And while the birth place and presence of heart disease may support a positive diagnostic test, these criteria do not appear to distinguish between those likely to have reactivity with one or more recombinant proteins in the selected panel and those who do not react. However it is clear that conventional serological tests fail to detect a substantial number of individuals, many with antibodies to multiple *T. cruzi* antigens. It is noteworthy that screening of sera with a parasite lysate also routinely fails to detect sera that exhibit reactivity to multiple recombinant *T. cruzi* proteins. The set of 4 most frequently recognized proteins detected all 7 of the discordant positive samples as well as 13 of the 15 discordant negative or negative samples that reacted with at least 1 protein. Expanding the panel to the 7 proteins that detected all of the seropositive samples (see above) allowed us to detect all of these 15 questionable “negative” samples.

### Monitoring treatment efficacy

**Figure 5:**
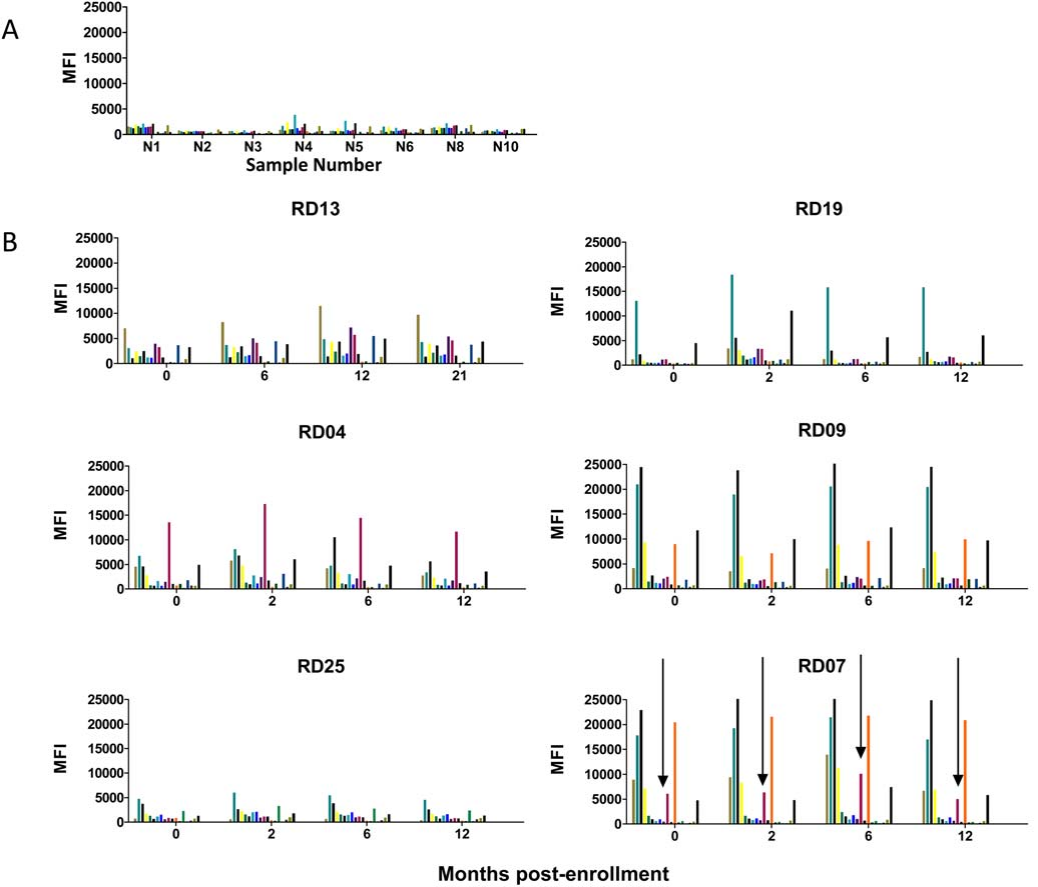
Stability of serological responses over time. The MFI of sera to a panel of 16 recombinant proteins (top 16 in Table 1), GFP negative control protein and *T. cruzi* lysate for a total of 18 measurements (bars) are shown for each serum. A) Reactivity of a set of 8 known negative sera. B) Stability of unique pattern of antigen activity for 6 seropositive subjects assayed at 4 time points over 12–21 months. Arrows in lower right panel (RD 07) indicate that detection of protein “8” (paraflagellar rod protein) which distinguishes the pattern of reactivity of serum RD07 from that of the similar RD09.

**Figure 6:**
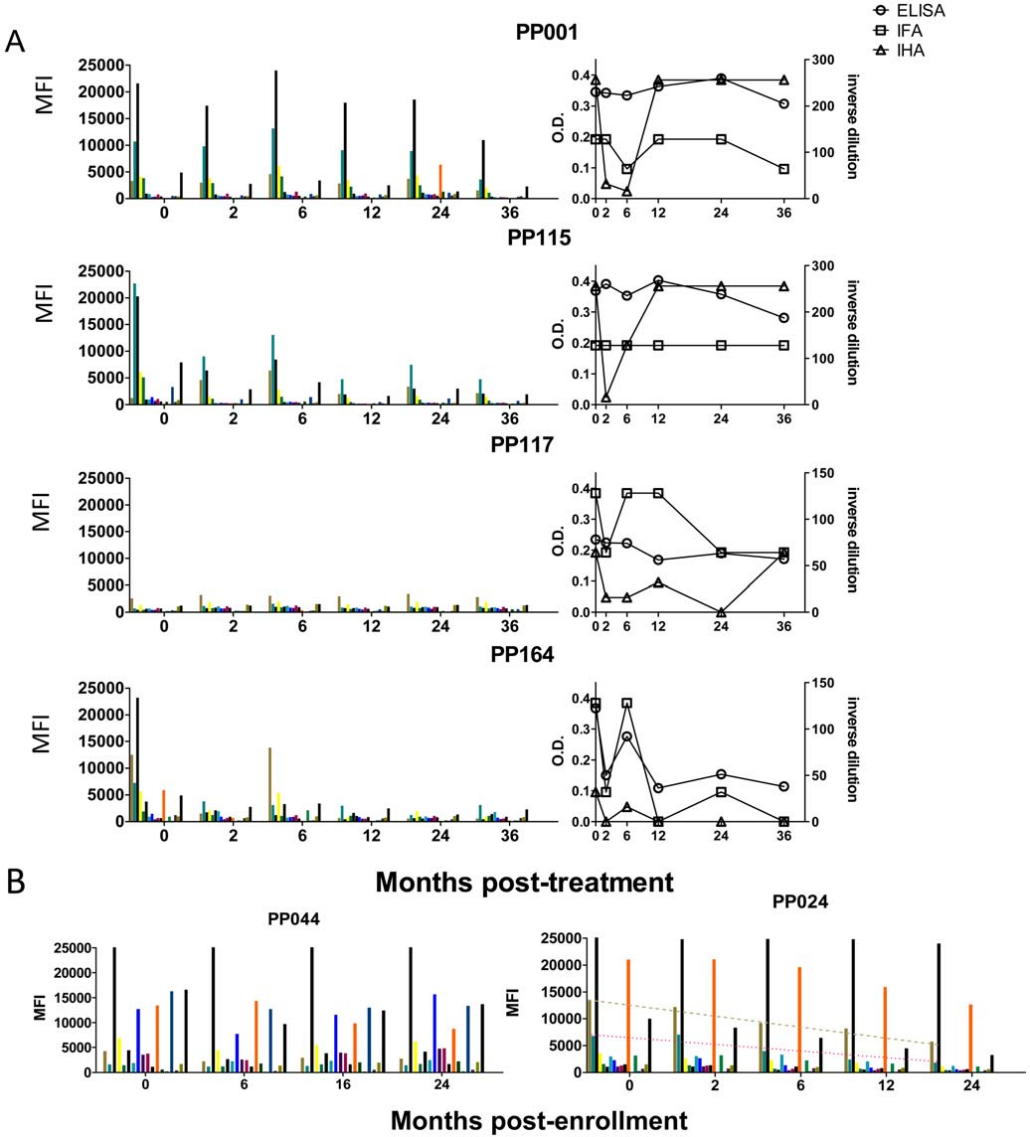
Effect of benznidazole treatment on serological responses in chronically infected subjects. The MFI of sera to a panel of 16 recombinant proteins (top 16 in Table 1), GFP negative control protein and *T. cruzi* lysate for a total of 18 measurements (bars) are shown for each serum. A) Change in pattern of reactivity over 36 months post- benznidazole treatment in 4 subjects using both the multiplex serologic assay (left) and conventional serology (right). B) Sera from 2 benznidazole-treated subjects exhibiting no evidence of change in multiplex assay for 24 months post-treatment (left; PP044) and changes in reactivity to selected recombinant proteins (right; PP024).

There is a pressing need for a means to assess treatment efficacy in Chagas disease so we next used the mulitplex assay to monitor changes in serology over time in subjects treated with benznidazole (BZ). Representative data from a set of 16 non-endemic normals (**Figure 5A**) demonstrates the background level of detection of responses in uninfected individuals, displayed as the MFI for each protein. To establish the stability of serological responses over time in the absence of treatment, serial serum samples were obtained from chronically infected, seropositive subjects, all without clinical disease; a representative set of 6 subjects screened at 4 times points for up to 21 months is shown in **Figure 5B**. Each subject exhibits a distinct pattern of serological responses and both the pattern and the potency of those responses are remarkably stable over time. In contrast, a representative set of 4 (from a total of 38) subjects followed for up to 36 months after treatment with BZ shows that some subjects exhibit a post-treatment decrease in the strength of responses to most *T. cruzi* antigens tested (**Figure 6A**). In many cases this fall is evident by 2 months post-treatment (e.g. PP001, PP115, PP164) and is followed by a transient increase at 6 months. Interestingly, this early drop in antibody levels following treatment is also sometimes evident, although less consistently so, with conventional serological tests, particularly with indirect hemaglutination (**Figure 6A**). Subject PP117 has borderline positive serology in both the multiplex and the conventional serologic assays and is representative of a case in which documenting changes following treatment would be difficult. **Figure 6A**). **Figure 6B** presents 2 other patterns of responses following treatment. PP044 shows essentially no change in the pattern or potency of antibody responses up to 24 months post-treatment. Subject PP024 is similar in that responses to the several prominently detected proteins are relatively stable over time. However the MFI reading for numerous other antigens falls consistently over the 24 month monitoring period. Thus, although it might take more time and additional assays to determine treatment efficacy in these two subjects, a preliminary assessment would be that treatment failed in the case of PP044 but was successful for subject PP024. A separate manuscript describing in greater detail both the cellular and serological responses to *T. cruzi* flowing BZ treatment is currently in preparation (Laucella, et al., in preparation).

## Discussion

The poor quality of diagnostics for *T. cruzi* infection is a major impediment to coping with a disease that affects as many as 20 million people. Without quality diagnostics, the statistic of the disease burden is at best a guess, the ability to conclusively identify who should be treated, or should be allowed to donate blood or tissues is greatly compromised and the effectiveness of interventions to limit transmission or drugs to treat those infected is impossible to determine with any certainty.

In the early stages of *T. cruzi* infection, parasites can often be detected in blood. However, as immunity develops, even amplification techniques such as xenodiagnosis, hemaculture, and PCR, despite being repeated multiple times, routinely fail to detect infection [12],[18],[32],[33]. Consequently, determination of infection status is largely dependent on the consensus results of multiple tests with different formats (e.g. ELISA, indirect fluorescent antibody, indirect hemaglutination, complement fixation). However the unreliability of these tests is well documented [9],[10],[11],[12],[13],[14],[15],[16],[17],[18],[19]. Many of these tests, including one recently licensed by the United States Food and Drug Administration for use as a blood screening test in the U.S. [20], use crude or semi-purified parasite preparations derived from parasite stages present in the insect vector but not in infected humans. Recently, a number of recombinant parasite proteins or peptides have also come into limited use for diagnosis [21],[22],[34],[35].

A subject whose serum is consistently positive on multiple of the currently used tests is relatively easily determined to be infected. But the infection status of individuals positive on only one test (as used in blood bank screening) is unclear and detection of parasites in subjects who are negative using multiple conventional serologic tests [10],[16],[17],[18],[36] or who are positive by alternative but not widely available serological tests [13],[19] is not uncommon. Furthermore, currently available tests are inadequate for monitoring treatment efficacy [37],[38],[39] and thus may also give inaccurate measurements of the effectiveness of other interventions.

With these deficits in mind, we set out to identify parasite proteins that would more effectively detect *T. cruzi* infection and provide a tool for monitoring changes in infection status over time. Development of a repository of nearly 1500 *T. cruzi* genes cloned into Gateway entry vectors provided a relatively straightforward approach to producing a large number and diversity of *T. cruzi* proteins appropriate for high-throughput screening of diagnostics. Adding the targeted approach of selecting proteins documented for high level expression in trypomastigote and amastigote stages of *T. cruzi* allowed us to also focus on the proteins that would be predicted to elicit the strongest antibody response in infected humans. The Luminex-based multiplex bead array system permitted us to screen many proteins simultaneously with very low requirements for serum. The production of histidine-tagged proteins also made it relatively uncomplicated to attach the recombinant proteins to Luminex beads. This latter point is not trivial as the proteins could be coupled to the assay beads directly from the urea-based denaturing lysis buffer without the requirement of movement to a non-denaturing buffer, wherein many of the proteins precipitated. The strong response detected using proteins prepared in this way suggests either that natively folded proteins are not required for the detection of these antibodies, or that re-folding of the proteins attached to the Luminex beads during buffer exchange resulted in the formation of native conformational epitopes.

In addition to its utility for screening of a large number of proteins, the Luminex system also excels as a platform for multiplex analysis of antibodies to a relatively large set of targets. We were restricted in this work by the number of Luminex bead sets manufactured with Ni+2 and thus sought to identify a maximum of 16 independent *T. cruzi* proteins that gave informative results from a large set of human sera. The ultimate panel selected by the screen included at least one protein previously identified as a potential diagnostic, the mitochrondrial HSP-70 [40]. It is possible that other proteins revealed in our screen have been studied previously. However since the identity of some of these previously assayed proteins is somewhat cryptic [21] and few have been associated with annotated genes in the sequenced *T. cruzi* genome, this possibility is difficult to evaluate. Also, over half of the antigens selected in our screen were among the 50 most abundant proteins in the trypomastigote and amastigote proteomes [31]. Two hypothetical proteins and 2 proteins unique to *T. cruzi* among the sequenced kinetoplastids, including 2 fragments from the very large and multicopy dispersed gene family protein, were among the proteins selected. Proteins that are unique to *T. cruzi* could be particularly useful in a serological screen as they are absent from *Leishmania*, one of the potentially confounding infections in terms of diagnosis of *T. cruzi*. However the dispersed gene family fragments were among the worst performers in the large scale screen – with only 5–9% of all confirmed positive sera having detectable antibodies to these. Similarly, other gene family proteins, including trans-sialidases, mucins and mucin-associated proteins (MASPS) were part of the screen but failed to make even the initial selection cuts in our assays, presumably because only a small fraction of their diversity would be represented in the recombinant proteins screened.

A multiplex approach like the Luminex also provided a more detailed examination of responses than is possible using a single target consisting of either an individual protein or a protein/peptide mixture. Each individual was seen clearly to have a distinct pattern of responses to the protein panel and this pattern was impressively stable over time (several years). This is both interesting scientifically and serves as further validation of the quality and consistency of the data generated using this multiplex methodology. This heterogeneity of responses to pathogens among individuals appears to be more the norm than the exception, as similar results have been reported for individuals infected or immunized with viral (vaccinia), bacterial (*Francisella tularensis*) and protozoal (*Plasmodium falciparum*) pathogens [41],[42],[43]. Thus serodiagnostics in general are likely to need to move toward multiplex assays, as single antigens that are recognized by all individuals infected by any pathogen appear to be rare [41].

The ability to simultaneously and independently assess antibody responses to multiple targets was instrumental to our success in addressing the issues of the detection of serological responses in subjects who are negative by conventional serology and the relatively rapid detection of changes in selected responses following drug treatment. The multiplex assay detected 100% of 121 samples consistently positive by conventional serology, and 100% of samples positive on 2 out of 3 conventional tests. In addition, however, we also detected antibodies specific for one or more recombinant proteins in 18 of 33 subjects judged as negative by conventional serology. Other investigators have documented cases of conventional seronegative subjects being seropositive on alternative tests or even parasite positive [10],[13],[16],[17],[18],[19],[36] although these previous reports of “infected seronegatives” have been somewhat anecdotal – presumably because investigators rarely screen for parasites in seronegative subjects. However in some studies parasite-positive conventional seronegatives are very well documented. For example Picka et al. [18] reported on one subject who was negative by up to 5 replicates of 4 different conventional serological tests yet was positive by a combined hemaculture-PCR approach. The multiple examples of the failure of conventional serology to detect infection, in combination with the well-documented unreliability of parasitological tests, supports the conclusion that individuals who are seropositive in our multiplex assay are likely to be infected with *T. cruzi*. This conclusion is further supported by on-going studies demonstrating *T. cruzi* –specific T cell responses in subjects who are negative by conventional serology but positive in our multiplex assays (Postan, et al. in preparation). Without more sensitive parasitological tests we cannot conclusively determine if the subjects who are negative by conventional serology but positive in our multiplex assay are infected or possibly “exposed” but not still infected with *T. cruzi*. And without additional extensive validation, we cannot exclude the possibility that other infections or immunological conditions resulted in some of the multiplex positive responses, although standard clinical analysis failed to detect other complicating infections in these subjects. However, especially for subjects who have antibodies to up to 8 different recombinant *T. cruzi* proteins and were born in endemic areas and/or have evidence of heart disease, it is reasonable to conclude that they are indeed infected with *T. cruzi* despite their negative results with conventional serologic assays. Overall these studies support the already documented conclusion that current serological tests can misdiagnose infection – perhaps to a significant extent.

A second issue we addressed using the multiplex serological assay for *T. cruzi* infection was that of efficacy of therapeutic treatment. Because most subjects are negative by parasitological assays prior to treatment (making a negative result after treatment uninformative) and remain positive by conventional serology for extensive periods of time after treatment, assessing whether treatment actually achieved cure, has been problematic. When combined with other evidence of treatment failures and the adverse effects of the drugs, the absence of a method to detect treatment efficacy has resulted in a very low rate of treatment in chronic Chagas disease. This absence of a reliable and timely test for treatment efficacy is also a major impediment to the development and testing of new drugs – an area that has been at a virtual standstill for decades.

Herein we show that the multiplex assay using the selected set of recombinant proteins can detect significant changes in antibody levels, in some cases as early as the first post-treatment assay point (2 months post-treatment completion). These changes are not evident in all cases – an outcome that is not surprising given that treatment failure is common [44]. Our ability to assess responses to multiple targets on an individual basis appears to be crucial to the success of detection of serologic changes following treatment, as similar changes are not consistently observed using conventional serologic tests. Previous studies have suggested that various recombinant antigens may provide better assessment of treatment efficacy relative to conventional serology [37],[45]. Further studies of a large set of treated subjects, using both multiplex serology and cellular immune responses support the hypothesis that these immunological markers are effective indicators of treatment success or failure (Laucella, in preparation).

We define a set of diagnostic targets and an assay approach that we believe is a significant improvement upon current diagnostic tests for *T. cruzi* infection both for more consistently detecting infection and for assessing the effectiveness of treatment. Additional validation of these targets and the general methodology will require analysis of a larger set of subjects, a process that is currently on-going. Herein we have also not addressed the question of whether the antigens we identify would be useful throughout the wide endemic range for *T. cruzi*. Heterogeneity among different parasites strains in distinct regions could present a challenge. However here again this is a concern that a multiplex assay might rather easily address – it seems unlikely that all 16 proteins in our pool, most of which are abundant housekeeping proteins, would vary substantially among parasites in various regions. Furthermore, we have intentionally used a mixture of *T. cruzi* strains from geographically distinct regions as the source of genes encoding the proteins used in these studies, with the goal of capturing some of the heterogeneity that may exist in these proteins among various parasite isolates.

The problem of infection confirmation by detection of parasites or parasite products is likely to continue to be a roadblock to full acceptance of the results of this test, or any other, when they conflict with conventional serologic tests – despite the proven inadequacy of these “standard” tests. Currently there is no methodology that allows for the consistent detection of parasites or their products in chronically infected hosts. The well-documented failure of various PCR-based approaches indicates that even highly abundant *T. cruzi* sequences are insufficient to document active infection in the majority of individuals, in addition to confirming the very low level of parasite persistence in most individuals. If the million-fold amplification afforded by PCR is unable to consistently reveal persistent *T. cruzi* infection, it also seems that detection of other parasite “biomarkers” will be equally inadequate. One advantage of using host biomarkers such as pathogen-specific antibodies and T cells is that they are naturally and endogenously self-amplified in the course of immune recognition of the infection. Downsides of the Luminex system for multiplex analysis include the reagent expense as well as the requirement for specialized equipment to “read” the results. However, other multiplex platforms such as protein microarrays could be more cost conservative and require less infrastructure [41],[46]. Also, our results suggest that the number of proteins in the analysis could be reduced without substantial loss of sensitivity, and the possibility exists for additional improvements in sensitivity by the inclusion of *T. cruzi* proteins previously validated by others or that could be detected in additional screens like that described herein. At a minimum, these results begin to lay the groundwork for the removal of one of the major impediments to the development and effective implementation of treatments for *T. cruzi* infection.

## Supporting Information

Alternative Language Abstract S1 Translation of the Abstract into Spanish by Susana Laucella
(0.03 MB DOC)
