# Supplementary material for: High Throughput Selection of Effective Serodiagnostics for Trypanosoma cruzi infection
Source: PLoS Negl Trop Dis. 2008 Oct 8;2(10):e316. doi: 10.1371/journal.pntd.0000316 (PMC2556098; doi:10.1371/journal.pntd.0000316)
Supplement: Alternative Language Abstract S1 — Translation of the Abstract into Spanish by Susana Laucella (0.03 MB DOC) [file pntd.0000316.s001.doc]

Selección de alto rendimiento de serodiagnósticos efectivos para la infección con *Trypanosoma cruzi*

Resumen

Antecedentes: El diagnóstico de la infección por *Trypanosoma cruzi* a través de la detección directa del patógeno es complicado debido a la baja carga parasitaria en personas con infección persistente con el agente etiológico de la Enfermedad de Chagas. El diagnóstico de la infección por análisis serológico ha sido también deficiente debido, principalmente, a la falta de antígenos parasitarios bien caracterizados para la detección de anticuerpos contra el parásito.

Métodos: En este estudio, examinamos más de 400 proteínas recombinantes de *T. cruzi,* incluyendo proteínas seleccionadas al azar y otras altamente expresadas en estadios parasitarios presentes en el huésped mamífero, con el objetivo de determinar la habilidad de las mismas para detectar anticuerpos contra el parásito en el suero de personas con sospecha de infección o infección confirmada para *T. cruzi.*

Resultados: Un conjunto de 16 proteínas fueron identificadas e incorporadas en una matríz de esferas múltiples, las cuales detectaron el 100% de más de 100 sueros con serología positiva confirmada por ensayos convencionales. Esta matríz de proteínas también evidenció, consistentemente, amplias e intensas respuestas en muestras con resultados serológicos negativos o discordantes por ensayos serológicos convencionales. Cada suero presentaba un perfil reactivo distintivo pero muy estable. Este grupo de proteínas también resultó de utilidad para determinar la eficacia terapeútica durante el seguimiento del tratamiento etiológico de la enfermedad de Chagas crónica.

Conclusiones: Estos resultados extienden, sustancialmente, la variedad y calidad de blancos diagnósticos para la enfermedad de Chagas y ofrece una herramienta útil para determinar el éxito o fracaso del tratamiento específico para la infección por *T. cruzi.*
